# Pyroptosis and inflammasomes in diabetic wound healing

**DOI:** 10.3389/fendo.2022.950798

**Published:** 2022-08-05

**Authors:** Xingrui Mu, Xingqian Wu, Wenjie He, Ye Liu, Faming Wu, Xuqiang Nie

**Affiliations:** ^1^ College of Pharmacy, Zunyi Medical University, Zunyi, China; ^2^ Key Laboratory of Basic Pharmacalogy of Ministry of Education and Joint International Research Laboratory of Ethnomedicine of Ministry of Education, Zunyi, China

**Keywords:** pyroptosis, inflammasome, signaling pathways, NLRP3, diabetic wound

## Abstract

Diabetic wound is one of the complications of diabetes and is not easy to heal. It often evolves into chronic ulcers, and severe patients will face amputation. Compared with normal wounds, diabetic wounds have an increased proportion of pro-inflammatory cytokines that are detrimental to the normal healing response. The burden of this disease on patients and healthcare providers is overwhelming, and practical solutions for managing and treating diabetic wounds are urgently needed. Pyroptosis, an inflammatory type of programmed cell death, is usually triggered by the inflammasome. The pyroptosis-driven cell death process is primarily mediated by the traditional signaling pathway caused by caspase -1 and the non-classical signaling pathways induced by caspase -4/5/11. Growing evidence that pyroptosis promotes diabetic complications, including diabetic wounds. In addition, inflammation is thought to be detrimental to wound healing. It is worth noting that the activation of the NLRP3 inflammasome plays a crucial role in the recovery of diabetic wounds. This review has described the mechanisms of pyroptosis-related signaling pathways and their impact on diabetic wounds. It has discussed new theories and approaches to promote diabetic wound healing, as well as some potential compounds targeting pyroptosis and inflammasome signaling pathways that could be new approaches to treating diabetic wounds.

## Introduction

Diabetes is a metabolic disease caused by a variety of etiologies, and the number of people suffering from type 2 diabetes mellitus (T2DM) is growing every year (1). By 2045, the number of people with diabetes is expected to exceed 700 million (7.8% of the global population) (2). Diabetes has over 100 complications, making it the most well-known disease. These complications include cardiovascular disease, peripheral neuropathy, chronic renal failure, stroke, and diabetic wounds or ulcers (3). Diabetic wounds are a condition that affects about 20% of patients with diabetes (4). Diabetic wounds are characterized by impaired healing responses, prolonged inflammation, and reduced epithelialization kinetics in diabetic patients (5).

Wound healing is a physiological response to structural tissue injury, which includes skin damage. Wound healing is a multi-stage process that includes hemostasis, inflammation, proliferation, and remodeling (6). Diabetic wounds heal slower than normal wounds due to the production of pro-inflammatory mediators, ischemia induced by microvascular problems, particular metabolic deficiencies, and decreased production of healing-related components, among other causes (7). As a result, diabetic wounds have a longer course and more complex mechanisms than normal wounds, affecting patients’ morbidity, mortality, and quality of life significantly (8).

Pyroptosis, an type of programmed cell death, is usually associated with inflammatory responses (9). According to new research, cell death pathways include apoptosis, necroptosis, autophagy, ferroptosis, cuproptosis, pyroptosis, and necrosis, and the regulators and effectors of these pathways remain promising therapeutic targets (10). When germs, pathogens, or endotoxins stimulate cells, the caspase family becomes active, triggering pyroptosis, also known as inflammatory cell death. As a result of this process, cell swelling, cell membrane pore formation, cell membrane rupture, inflammasome activation, and finally the release of cell contents and inflammatory mediators all occur, culminating in severe inflammatory responses (11, 12).

In conclusion, this review summarizes the research progress on the relationship between pyroptosis and diabetic wounds. The primary purpose of this review is to clarify the mechanism of pyroptosis-related signaling pathways and their impact on diabetic wound, explore new therapeutic approaches and identify potential therapeutic targets.

## Pyroptosis and inflammasome

Cell death dependent on caspase-1 was originally called apoptosis (13). In 2000, Cookson introduced the term “pyroptosis”. Pyroptosis differs from apoptosis in the morphological features and inflammatory nature of cell death (14, 15). Pyroptosis is involved in the pathogenesis of various diseases, such as tumors, cardiovascular diseases, COVID-19, diabetes and its complications. The mechanism of pyroptosis is regulated by many protein or protein complexes, such as caspase, inflammasome, and Gasdermin. Typically, caspases exist in an inactive precursor form, a pro-caspase. After activation by the inflammasome, caspases cleave Gasdermin and generate a hydrophilic C-terminal domain and a lipophilic N-terminal domain. The N-terminal lipophilic domain oligomerizes and binds to the cell membrane to form a pyroptotic pore, ultimately triggering pyroptosis.

Caspases, a group of proteases with similar structures present in the cytoplasm, are known to drive apoptosis or pyroptosis and play a key role in programmed cell death and inflammation (16). Mammalian caspases fall into two broad categories: apoptotic and inflammatory caspases. Among them, caspase-1, -4, -5, -11, and -12 belong to the family of inflammatory caspases, which are closely related to pyroptosis (17). According to the latest research, the signaling pathways of pyroptosis can be split into four kinds based on different activation modalities: canonical inflammasome signaling pathways (caspase-1), non-canonical inflammasome signaling pathways (caspase-4/5/11), and pyroptotic pathways depending on caspase-3 and caspase-8 (18–20).

The inflammasome is a multi-protein complex assembled by intracellular pattern recognition receptors (PRRs) and is an essential part of the innate immune system (21, 22). Damage-associated molecular patterns (DAMPs), endogenous molecules released by the body’s cell death, namely endogenous danger signals, originate from immune cells activated by damaged or necrotic tissue. Pathogen-associated molecular patterns (PAMPs) are ligand receptors that PRRs recognize and bind, mainly referring to some highly conserved molecular structures shared on the surface of pathogenic microorganisms, such as lipopolysaccharide of G-bacteria. Generally, both PAMPs and DAMPs induce inflammasome activation *via* PRRs. Inflammasomes can recognize PAMPs or DAMPs and recruit and activate the pro-inflammatory protease caspase-1, thereby inducing cell death under pathological conditions of inflammation and stress. It is known that this process can promote the maturation and secretion of pro-IL-1β and pro-IL-18 during innate immune defense (23–25).

Current research indicates that there are five common inflammasomes, including NLRP1, NLRP3, NLRC4, Pyrin, and AIM2 (26). The NLRP3 inflammasome, in particular, has been widely investigated and is involved in a number of diseases, including type 2 diabetes and diabetic wounds (22, 27). NLRP3 may promote interaction with the pyrin domain (PYD) in ASC in response to immune activators (e.g., PAMPs, DAMPs), other exogenous invaders, or environmental stimuli. Subsequently, the caspase recruitment domain (CARD) of ASC binds to the CARD domain on pro-caspase-1 to generate the NLRP3 inflammasome. The creation of this complex causes pro-caspase-1 to self-cleave, resulting in an active caspase-1 p10/p20 tetramer, and the maturation of the pro-inflammatory cytokines IL-1β and IL-18 from their immature “pro” versions.

**Figure 1:**
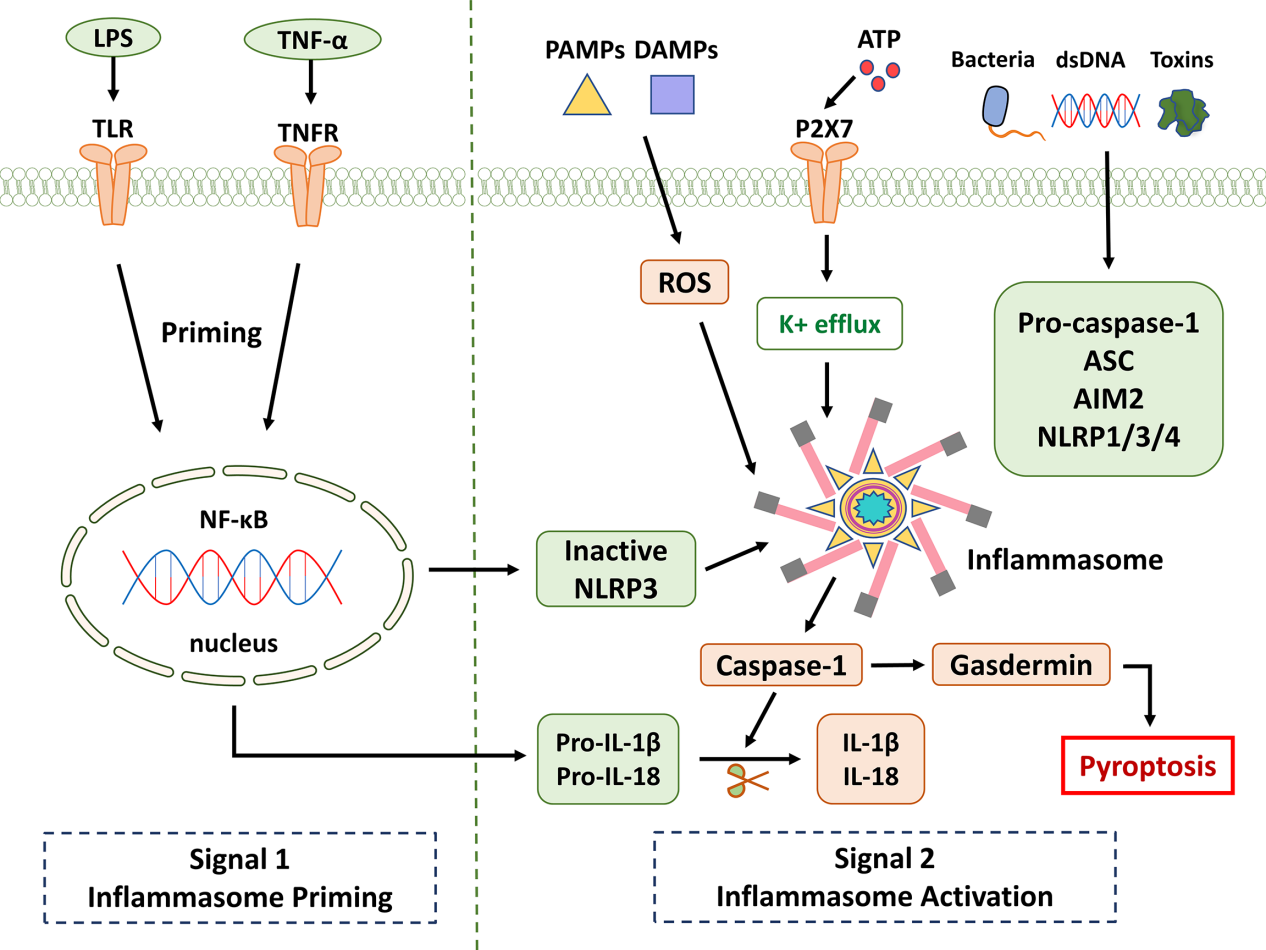
NLRP3 inflammasome signaling pathway. It is known that the activation of NLRP3 is divided into two key steps: priming and activation. Signal 1 (priming; left): When pathogens and their products, injury, stress, and other signals stimulate cells, TLR, TNFR, and other receptors can be activated. NF-κB is activated through different pathways, which in turn promotes NLRP3, pro-IL-1β, and pro-IL-18. Provide a material basis for the activation and function of NLRP3 inflammasome. Signal 2 (activation; right): Multiple upstream signaling events are activated by PAMPs and DAMPs. These include K^+^ efflux, reactive oxygen species (ROS) production, etc. Inflammasome formation activates caspase-1, which cleaves pro-IL-1β and pro-IL-18. Gasdermin is also cleaved and inserted into membranes, forming pores and inducing pyroptosis.

Activation of the NLRP3 inflammasome appears to proceed in two steps. The first step involves priming or initiation signals, and many PAMPs or DAMPs are recognized, leading to the activation of NF-κB-mediated signaling that upregulates the transcription of inflammasome-related components, including inactive NLRP3, pro-IL-1β, and pro -IL-18. The second step is the oligomerization of NLRP3 and the subsequent assembly of NLRP3, ASC, and pro-caspase-1 into a complex (28). Based on the available evidence and research results, pro-caspase-1 conversion to caspase-1 is accompanied by the release of mature IL-1β and IL-18. Furthermore, triggering PAMPs and DAMPs causes the creation of ROS, which promotes the assembly and activation of the NLRP3 inflammasome (**Figure 1**). Therefore, as the center of the inflammatory response, the NLRP3 inflammasome could be a potential therapeutic target for inflammatory illnesses. However, non-canonical inflammasome activation pathways mediated by mouse caspase-11 or human caspase-4/5 are independent of inflammasome activation. Pyroptosis was also successfully induced in response to intracellular lipopolysaccharide (LPS) detection (29). ASC (also known as PYCARD) is a protein containing pyran and CARD domains that aid in inflammasome assembly (30).

The six members of the gasdermins are Gasdermin A–E and DFNB59 (31). Except for DFNB59, other components have been reported to be associated with the pyroptotic process. A peptide linker connects an N-terminal domain (effector domain that creates the transmembrane pore) to a C-terminal domain (with auto-inhibitory effects). Among gasdermins, gasdermin D(GSDMD) and gasdermin E(GSDME) are the most well-characterized in terms of activation and function (32). Gasdermin family members are mainly expressed in skin, gastrointestinal tract, and immune cells to actively eliminate infected cells through pyroptosis (33, 34). GSDMD is a substrate of caspase-1, a component of the inflammasome responsible for executing pyroptosis and secreting mature IL-1β (35). When caspase cleaves the gasdermin-N domain free, it binds to the lipids on the cell membrane and encourages the creation of pores, which causes the membrane to rapidly lose its integrity and allow the contents of the cell to flow out. Pore-forming activity and pyroptosis are therefore reduced in the absence of GSDMD activation (36). Of course, two critical steps in pyroptosis are inflammasome activation and GSDMD cleavage. Recent studies have found that disruption of mitochondrial membrane potential (MMP) and reactive oxygen species (ROS) production is commonly associated with macrophage pyroptosis (37).

## Signaling pathways of pyroptosis

### Canonical inflammasome signaling pathway (Caspase-1)

**Figure 2:**
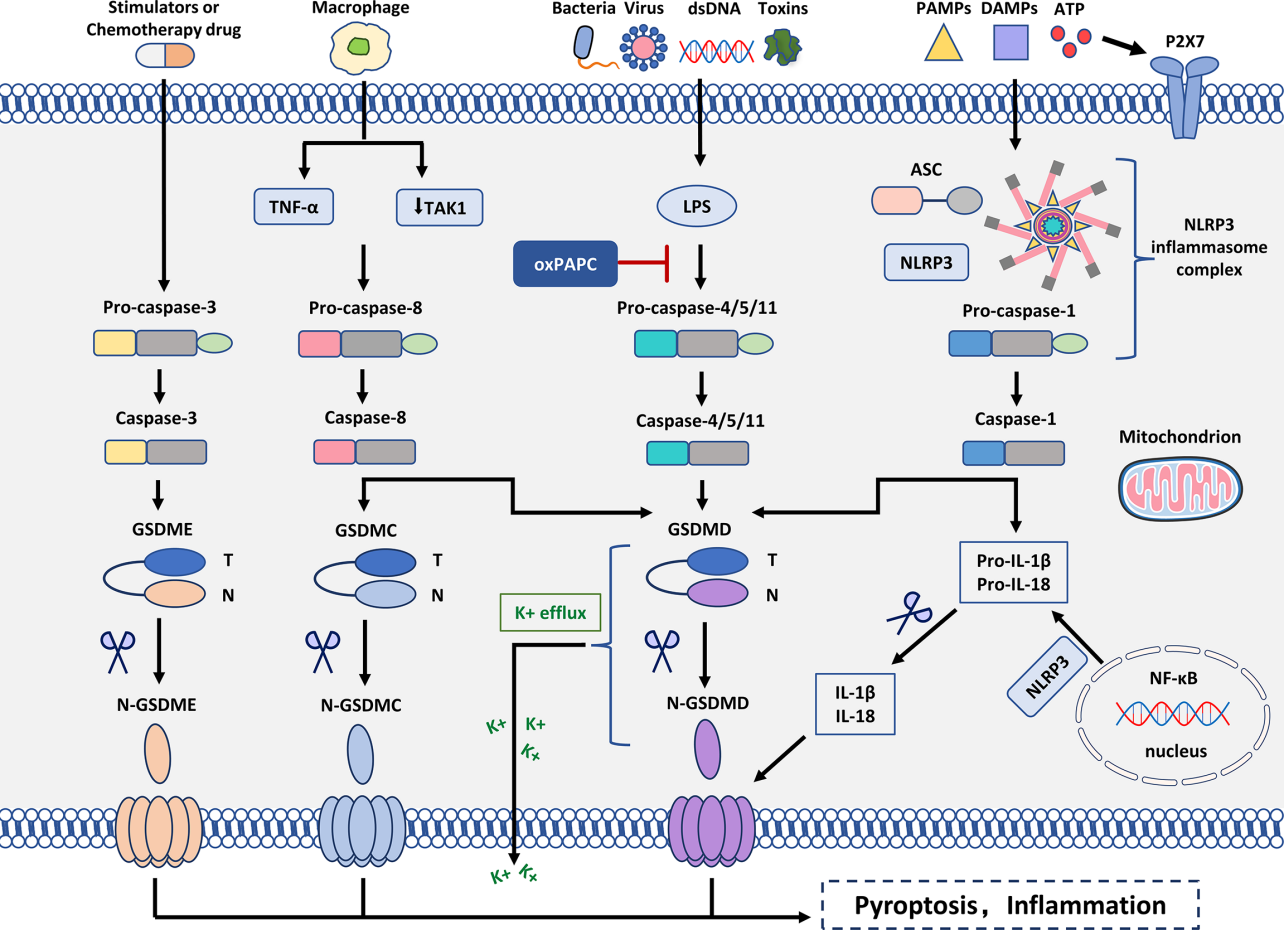
Molecular mechanism of pyroptosis. In the canonical inflammasome signaling pathway, PAMPs, DAMPs, and extracellular ATP are stimulated by intracellular signaling molecules and assembled with pro-caspase-1 and ASC to form inflammasomes and activated caspase-1. N-GSDMD perforates cell membranes by forming non-selective pores. In addition, IL-1β and IL-18 were secreted in the pores formed by N-GSDMD. In the non-canonical inflammasome signaling pathway, intracytoplasmic LPS activates caspase-4/5/11, which triggers pyroptosis by cleaving GSDMD. However, oxPAPC competes with LPS for binding to caspase-4, thereby inhibiting pyroptosis. Cleavage of GSDMD leads to K+ efflux that ultimately mediates the assembly of the NLRP3 inflammasome. Moreover, cleavage of GSDMD leads to cleavage of pro-IL-1β and pro-IL-18. In the caspase-3-mediated pathway, active caspase-3 cleaves GSDME to form N-GSDME, inducing pyroptosis. In the caspase-8-mediated pathway, inhibition of TAK1 induces the activation of caspase-8, which cleaves GSDMD, leading to pyroptosis. In addition, under hypoxia, TNF-α activates caspase-8 after apoptosis to pyroptosis, which can regulate the transcription of GSDMC.

In most cases, the formation of the inflammasome requires pattern recognition receptors (PRRs) as sensors, the adaptor protein ASC (CARD-containing apoptosis-associated speck-like protein) and caspase1 (38). The assembly of inflammasomes triggers the hydrolysis of inactive pro-caspase-1 to active caspase-1, which converts the cytokines pro-IL-1β and pro-IL-18 into mature and bioactive IL-1β and IL-18, respectively (39, 40). Caspase-1 cleaves GSDMD, which is involved in membrane pore formation. Membrane pores cause the production of inflammatory molecules, including IL-1β and IL-18, which causes the cells to expand and eventually develop into pyroptosis (**Figure 2**).

Many multifactorial diseases, such as gouty arthritis, atherosclerosis, and type 2 diabetes, are exacerbated by NLRP3-mediated inflammation. PAMPs/DAMPs that activate NLRP3 include hyperglycemia, fatty acids, protein aggregates, and extracellular ATP, among many others (41, 42). The mechanism of activation of the NLRP3 inflammasome is clearly described in paragraph 2 (**Figure 1**). The inflammasome effector cytokines IL-1β and IL-18, due to NLRP3 activation, are major effector molecules that exacerbate these diseases. As a result, maintaining optimal cellular homeostasis and health requires fine-tuning NLRP3 inflammasome activity (43).

### Non-canonical inflammasome signaling pathway (Caspase-4/5/11)

Mouse caspase-11 and its human orthologs caspase-4 and -5 are involved in non-canonical inflammasome signaling, and investigations of mouse caspase-11 have led to various findings concerning their immunological role (44). The TLR4/MD2 complex identified the pathogen-associated molecular pattern to activate inflammatory responses as lipopolysaccharide (LPS), a significant component of Gram-negative bacteria’s outer membrane (45). Myeloid differentiation factor 2 (MD2) is a key mediating protein required for the dimerization/activation of TLR4 (Toll-like receptor 4). LPS activates pro-caspase-4/5/11, and pro-caspase-4/5/11 initiates pyroptosis-mediated cell death (46). Caspase-4/5/11 binds to LPS and cleaves the 53-kDa precursor form of the GSDMD protein molecule, resulting in the formation of the N-terminus of the mature GSDMD p30 fragment. Thus, the formation of pores in the cell membrane results in the release of IL-1β and IL-18 from the cells and induction of pyroptosis (47, 48). Caspase-4/5/11 can also stimulate NLRP3-mediated caspase-1 initiation and IL-1β/IL-18 *via* GSDMD cleavage. GSDMD has been identified as an important downstream component of both canonical and non-canonical inflammasome pathways involved in pyroptosis (49, 50) (**Figure 2**).

### Caspase-3-dependent pyroptosis pathway

Caspase-3, an apoptosis-executing protein, is the main protein responsible for the cleavage and activation of GSDME. In addition to GSDMD, GSDME also plays a crucial role in pyroptosis (51). Caspase-3 is activated by the mitochondrial and death receptor pathways and cleaves GSDME to form GSDME-N fragments, which lead to plasma membrane pore formation, cell swelling, and pyroptosis (52). GSDME protein molecules can be cleaved into N-terminal and C-terminal fragments by caspase-3. GSDME-N components are equivalent in function and purpose to GSDMD-N components (53). GSDME directly induces tumor cell pyroptosis through Caspase-3 and indirectly acts on T lymphocytes through Granzyme B, acting as a tumor suppressor gene (54). Furthermore, when chemotherapeutic drugs activate Caspase-3, primary human cells exhibit GSDME-dependent pyroptosis, providing new insights into cancer chemotherapy (54). These findings indicate that different caspase substrates, rather than activated caspases, influence the type of cell death induced.

### Caspase-8-dependent pyroptosis pathway

Recent research has revealed unexpected roles for caspase-8’s enzymatic activity and scaffold function in inflammasome activation and pyroptosis induction (55). The activation of the ASC-caspase-1 inflammasome is caused by the production of catalytically inactive caspase-8, leading to GSDMD-mediated pyroptosis (56). In the presence of inhibitors, activation of caspase-8 cleaves GSDMD leading to pyroptosis. More research is needed to see if caspase-8 can directly cleave GSDMD or if other intermediary substrates, other than caspase-1/11, are necessary to produce the pore-forming p30 subunit (57). However, pure active caspase-8 has been demonstrated to cleave recombinant mouse GSDMD creating p30 pore-forming fragments (58). Following caspase-8 activation in tumor cells, GSDMC has been found to mediate tumor necrosis. The pyrogenic cell death mediated by GSDMC/caspase-8 provides essential insights into the pyroptotic pathway in cancer cells (59). Based on the current research results, the mechanism of pyroptosis induced by caspase-8 is not clear enough, and the research on its mechanism is a direction worthy of attention in the future.

## Role of pyroptosis and inflammasomes in wound healing

Acute wound healing includes four stages: hemostasis, inflammation, proliferation, and remodeling (60). Excessive or long-term inflammation is one of the main characteristics of chronic wounds, since this condition negatively affects wound healing and leaves scars behind (61). The NLRP3 inflammasome is expressed in epithelial tissues, such as skin. As the first line of defense against external threats, it can participate in the skin’s innate immunity (62, 63).

The role of the NLRP3 inflammasome in the early stages of cutaneous wound healing is increasingly being investigated. Results of studies have demonstrated the effect of mulberry leaf and fruit extract (MLFE) on skin wound healing and the involvement of the NLRP3 inflammasome (64). Concurrently, research has revealed the function of the NLRP3 inflammasome in the proliferative and remodeling phases of wound healing (65). A combination of mulberry leaf and fruit extract (MLFE) provided better anti-obesity and anti-inflammatory benefits than mulberry leaf extract alone. Under obese conditions, the expression of the NLRP3 inflammasome and its associated markers (pro-caspase-1, IL-1β-precursor and IL-1β-mature) is higher than basal levels. Among them, the NLRP3 inflammasome is inhibited during the inflammatory phase of skin wound healing (66). The results showed that the addition of MLFE reduced body fat mass, fasting blood glucose levels, blood lipid levels, and hepatotoxicity. Therefore, body fat mass and fasting blood glucose may be potential indicators of delayed wound healing in obese patients. Although the exact activator or mechanism of the NLRP3 inflammasome is not known, it can be determined that MLFE normalizes the levels of the NLRP3 inflammasome and suppresses skin inflammatory responses during the early stages of wound healing in obesity (67). Deletion of the NLRP3 inflammasome will result in a decrease in proinflammatory cytokines such as IL-1β and TNF-α and delayed angiogenesis (68). MLFE may have potential therapeutic value in treating obesity and obesity-related complications, and NLRP3 might be a promising target in the fight against obesity wounds since it may promote the early healing of wounds. However, this study focused on detecting NLRP3-related proteins and ignored the close relationship between the inflammasome and pyroptosis. The article only detects pro-caspase-1 is not enough. If the expression of GSDMD and caspase-1 protein is detected, it is enough to show that it is related to pyroptosis, which may be another way to explore.

DNA nanomaterials with distinctive spatial configurations are known as tetrahedral framework nucleic acids (TFNAs) (69). TFNAs have excellent biosafety with anti-inflammatory, antioxidant, anti-fibrotic, angiogenic, and skin wound healing activities with little toxicity (70). *In vitro* and *in vivo* research have uncovered that TFNAs increase corneal transparency, speed wound reepithelialization, and play a positive role in corneal epithelial wound healing (71). TFNAs are not only beneficial for corneal wound healing but also for skin wound healing. The results of the study showed that treatment with TFNAs accelerated the healing process of skin wounds and reduced scarring. This is the first report that nanophase materials with nucleic acid biological properties can accelerate wound healing and reduce scarring, suggesting that TFNA can be used to promote skin tissue regeneration (72).

In addition, studies have shown that TFNA can promote diabetic wound healing by accelerating processes such as angiogenesis, epithelialization, and collagen deposition. Through their antioxidant activity *via* the PI3K/Akt/Nrf2/HO-1 signaling pathway, TFNAs can protect endothelial cell function, reduce inflammation, and prevent oxidative damage. The PI3K/Akt/Nrf2/HO-1 signaling pathway is regulated by metformin and plays a key role in metformin-induced osteogenesis. Therefore, the use of TFNAs could help diabetic wounds recover faster (73). As various local or systemic diseases promote skin inflammation, fibrosis (the result of a dysregulated tissue repair response) begins to dominate the repair process when the intensity or duration of skin damage exceeds the ability of the tissue to repair. As a result, medicines that substantially prevent skin fibrosis while also reducing immunogenicity, inflammation, apoptosis, and pyroptosis are required. TFNA inhibits the pyroptotic pathway and reduces inflammatory cytokine levels and skin collagen content in studies. Both NLRP3 inflammasome and pro-caspase-1 levels were down-regulated after TFNA treatment, indicating that the inflammasome was reduced and the active form of caspase-1 was reduced, resulting in a subsequent down-regulation of N-terminal GSDMD levels. The results showed that TFNA has anti-inflammatory and anti-fibrotic abilities without cytotoxicity (74). In this study, proteins related to pyroptosis and inflammasome signaling pathways were detected, including pro-caspase-1, caspase-1, NLRP3, GSDMD, etc. In a nutshell, TFNAs have important research significance for skin wound healing and have been confirmed to be closely related to pyroptosis and inflammasome pathways.

The research progress of bioactive glass (BG) in soft tissue repair is relatively rapid, especially in wound healing. The findings suggest that BG may accelerate wound closure, granulation development, collagen deposition, and angiogenesis (75). The method used in this study is that BG inhibits endothelial cell pyroptosis and promotes wound healing by regulating the Cx43/ROS signaling pathway. Therefore, BG inhibits the activation of caspase-1 by the NLRP3 inflammasome, attenuate the perforation activity of GSDMD, and ultimately inhibit the pyroptosis of endothelial cells (76). In addition, BG inhibits the production of ROS while regulating the expression of connexin 43 (Cx43) (77). Subsequently, BG promotes the formation of blood vessels resulting in accelerated wound healing. The study also made further proofs showed that BG could reduce the expression of Cx43 and the level of ROS, which strongly suggested that BG could inhibit the pyroptosis of endothelial cells (78). It has been shown that inhibiting pyroptosis enhances angiogenesis in many animal models, implying that BG also enhances angiogenesis. It can be concluded that BG can promote wound healing by impeding pyroptosis through the Cx43/ROS signaling pathway. Even though this study did not directly regulate pyroptosis and inflammasome signaling pathways, it did confirm their close relationship. It inspires us is that fewer drugs and methods inhibit pyroptosis, but it can be achieved by modulating other signaling pathways.

While the wound healing process is complex and dynamic, the pyroptosis and inflammasome pathways also have complex connections. There is currently a lack of studies on the crosstalk between multiple signaling pathways in wound healing or on pyroptosis and changes in the NLRP3 inflammasome at different phases of wound healing. Therefore, further in-depth study of other factors in the wound healing process will also provide us with new insights into the mechanism of wound healing. New treatment methods such as new carriers and new Chinese herbal extracts have been evaluated and will be used in the clinic in the future. How to optimize individualized treatment strategies while improving chronic inflammatory and pyroptotic states during wound healing needs to be considered.

## Role of pyroptosis and inflammasomes in diabetic wound healing

Diabetic wound pathogenesis is complicated and involves numerous pathways. There is some evidence that suggests that the local hyperglycemic environment is the main factor leading to diabetic wounds, but recent research shows that factors such as oxidative stress damage, accumulation of advanced glycation end products (AGEs), and chronic inflammation are closely related to diabetic wounds (79, 80). Persistent inflammatory activation is the leading cause of chronic refractory diabetic wound, and an essential factor leading to diabetic foot ulcer (DFU), gangrene, amputations, and even the root cause of prolonged hospital stays and increased wound management costs (81).

Targeting the NLRP3 inflammasome, which plays a role in the pathophysiology of numerous inflammatory disorders, could be a potential target for enhancing diabetic wound healing. Neutrophils release extracellular traps (NETs) to defend against pathogens that induce tissue damage (82). NETs have been detected in diabetic wounds and have been associated with impaired healing processes, but the mechanism by which NETs pause wound healing and their role in promoting inflammatory dysregulation remain unclear (83). Overproduced NETs in diabetic wounds trigger NLRP3 inflammasome activation and IL-1β release in macrophages (84). Meanwhile, NETs up-regulates the levels of NLRP3 and pro-IL-1β through the TLR-4/TLR-9/NF-κB signaling pathway, triggering the production of ROS, and activating the NLRP3 inflammasome (85). Furthermore, in a diabetic rat model, NET digestion by DNase I reduced NLRP3 inflammasome activation, altered immune cell infiltration, and expedited wound healing (86).

In recent years, most studies have focused on the role of the inflammasome, especially NLRP3 and pyroptosis, on wound healing. The NLRP3 inflammasome has been characterized in a corneal epithelial wear model in which wound healing and non-regeneration of diabetic corneal wounds have been studied. Recent studies suggest that the NLRP3 inflammasome-mediated inflammation and pyroptosis contribute to the pathogenesis of diabetic keratopathy (DK) (87). NLRP3 is necessary for corneal wound healing and nerve regeneration in physiological circumstances (88). In diabetics, however, prolonged activation of the NLRP3 inflammasome causes corneal wound healing to be delayed and nerve regeneration to be hindered. In addition, inhibiting the AGEs/ROS/NLRP3 inflammasome axis genetically and pharmacologically greatly accelerates diabetic corneal epithelial wound closure and nerve regeneration (87). The study highlighted that ocular surface damage in diabetic mice might be related to ROS/NLRP3/Caspase-1/IL-1β signaling pathway (89, 90). Activation of the NLRP3 inflammasome by high glucose-induced P2X7R (purinergic ligand-gated ion channel 7 receptor) affects the pathogenesis of diabetic retinopathy (DR) (91). Therefore, it is known that NLRP3 inflammasome and pyroptosis play critical roles in DK and DR. In addition, NLRP3 and pyroptosis also have significant effects on diabetic wounds (**Table 1**).

**Table 1:** Compounds or Molecules Inhibiting the Pyroptosis Signaling Pathway for the Treatment and Management of Diabetic Wound.

Classification	Mechanism of Pyroptosis Inhibition	References
Glyburide	NLRP3/caspase-1/IL-1β/IL-18/ASC	(92)
*Bacillus subtilis (WB800N)*	NLRP3/caspase-1/IL-1β/IL-37/TLR2/ASC	(93)
Perforin-2	AIM2/GSDMD/IL-1β/ASC	(94)
*Bletilla striata* polysaccharide	NLRP3/IL-1β/TNF-α/ROS	(95)
Paeoniflorin	NLRP3/caspase-1/IL-1β/IL-18/TNF-α/ASC	(96)
Heparan sulfate	NLRP3/IL-1β/IL-18/TNF-α/ASC	(97)

NLRP3, NOD-like receptor family pyrin domain containing 3; AIM2, Absent in melanoma 2; ASC, CARD-containing apoptosis-associated speck-like protein; IL-1β, interleukin-1beta; IL-18, interleukin-18; IL-37, interleukin-37; TLR2, Toll-like receptor-2; GSDMD, gasdermin D; TNF-α, Tumour Necrosis Factor alpha; ROS, reactive oxygen species.

Glyburide is a commonly used sulfonylurea drug to treat type 2 diabetes (92). The capacity to suppress the NLRP3 inflammasome through a mechanism different from its ability to enhance insulin release from pancreatic beta-cells was recently discovered, and glyburide improves wound healing in diabetic mice (98). Cryopyrin/NALP3/NLRP3 is an essential component of the inflammasome triggered by PAMPs, DAMPs, and crystalline substances (99). Glyburide is the first chemical able to block PAMPs, DAMPs, and crystal-induced IL-1β production by acting upstream of cryoproteins. Inflammasome activity was persistent in macrophages (mø) isolated from diabetes and db/db mice wounds, which was associated with low expression levels of endogenous inflammasome inhibitors. Because a wound-conditioned medium activates caspase-1 and stimulates the production of IL-1β and IL-18 in cultured cells through a ROS-mediated route, soluble components in these wound biochemical conditions are sufficient to activate the inflammasome (100). Inhibiting inflammasome activity in wounds of db/db mice by topical application of pharmacological inhibitors improves wound healing. This treatment shifts from a pro-inflammatory state to a pre-healing state and increases pre-healing growth factor levels.

*Bacillus subtilis* is a probiotic that modulates immune responses and reshapes the gut flora (101). *Bacillus subtilis* (WB800N) has the ability to activate TLRs (Toll-like receptors) and enhance immune responses (102). In recent years, there has been evidence that diabetic wounds are associated with gut microbiota. Studies have shown that amoxicillin can reduce the alpha and beta diversity of the intestinal microbiota in mice, leading to intestinal microbiota disturbances, thereby alleviating diabetic wounds (103). It is also significant that *Bacillus subtilis* (WB800N) can relieve diabetic wounds by regulating Toll-like receptor-2 (TLR2) (93). TLR2 is a major innate immune response factor, and of immune response activation benefits diabetic wound healing (104). The results showed that *Bacillus subtilis* (WB800N) could increase the expression of TLR2, NLRP3, ASC and Caspase-1 in diabetic wound mice. However, the TLR2 antagonist SsnB could reduce the expression of TLR2, NLRP3, ASC and Caspase-1 in diabetic wound mice. NLRP3/ASC is required for Caspase-1 activation and pro-IL-1β cleavage to generate mature IL-1β. In conclusion, *Bacillus subtilis* (WB800N) promotes inflammatory response in diabetic wound mice by activating TLR2. This study only explored the NLRP3 inflammasome signaling pathway but lacked the detection of pyroptosis-related proteins. The authors believe that *Bacillus subtilis* (WB800N) promotes cell apoptosis, but it is actually more likely to be pyroptosis, which is worth exploring in the future.

Skin infections and the spread of *Staphylococcus aureus* (*S. aureus*) in mice are limited by perforin-2, an innate immune molecule against intracellular bacteria (105). The study showed the accumulation of *S. aureus* within the epidermal cells of DFU without clinical signs of infection due to significant inhibition of perforin-2 (106). Evidence from studies shows that *S. aureus* within the epidermis of DFU triggers AIM2 inflammasome activation and pyroptosis. From the results, increased induction of AIM2 inflammasome, ASC-pyroptosome, and IL-1β was found in non-healing DFU (107). The correlation of AIM2 with healing outcomes suggests that AIM2 has a central function in regulating the inflammatory response to DFU. There is evidence that the increase in IL-1β involved in pyroptosis is accompanied by an increase in the AIM2 inflammasome, resulting in the oligomerization of ASCs into pyroptosomes which then triggers the activation of pro-caspase-1 resulting in the cleavage of porins such as Gasdermin D, and the lysis of cells involved in inflammation (108). In patients with DFU, the inhibition of perforin-2, intracellular accumulation of *S. aureus*, and related blepharoptosis lead to the inhibition of wound healing and the persistence of inflammation (94). Intracellular *S. aureus* accumulates in the DFU epidermis as a result of perforin-2 suppression, triggering activation of the AIM2 inflammasome, which results in caspase-1-mediated IL-1β activation and proteolysis of the pore-forming gasdermin D processing. As a consequence of this cascade, holes are formed in the plasma membrane, opening up a pathway for pyroptosis and for the release of intracellular components. As a result, inflammatory mediators and accumulated intracellular S. aureus are released, leading to chronic inflammation and direct inhibition of wound healing. Further, it was determined that gasdermin D, one of the substrates of caspase-1, is also cleaved and activated by DFU (109). This study is the first to demonstrate that intracellular *S. aureus* can inhibit perforin-2 in DFU. Pyroptosis is the predominant form of cell death in DFU, but the possibility of other forms of cell death such as necroptosis, ferroptosis, and cuproptosis remains to be tested.

## Pyroptosis inhibitors for the treatment and management of diabetic wounds

A growing body of research suggests that *Bletilla striata* polysaccharide (BSP), the main active ingredient in Bletilla striata, promotes normal or diabetic wound healing (110). This is due to the fact that BSP improves diabetic wound healing by infiltrating fibroblasts and enhancing collagen synthesis in the skin wound tissue. Furthermore, BSP is suitable for medical applications, including wound dressings, hydrogels, tissue engineering scaffolds, and drug delivery vehicles (111–113). This study has demonstrated that the therapeutic effect of BSP on DFU is mediated by inhibiting HG-induced NLRP3 inflammasome activation in macrophages, which increases insulin sensitivity in endothelial cells (95). The results showed that the expressions of TXNIP, NLRP3, pro-caspase-1, cleaved-caspase-1, pro-IL-1β and cleaved-IL-1β were increased in diabetic skin wounds. However, BSP treatment resulted in decreased levels of some proteins, such as pro-caspase-1, cleaved-caspase-1, pro-IL-1β, and cleaved-IL-1β. In addition, the sensitivity of BSP to insulin was improved.

BSP has a protective effect on macrophages and can significantly reduce the amount of ROS produced by HG, thereby preventing macrophages from being induced by HG. In addition to this, IL-1β secretion and NLRP3 inflammasome activation were also inhibited (114, 115). Additionally, BSP is thought to play an important role in preventing HG-induced endothelial cell inactivation and ROS homeostasis imbalance (116). The results showed that BSP more effectively protected BMDMs (Bone marrow-derived macrophages) from the HG-induced ROS production, inhibited NLRP3 inflammasome activation and reduced IL-1β secretion. It also reduced the abnormal production of ROS in CMECs (cardiac microvascular endothelial cells) while maintaining cell viability (114). Macrophage infiltration and angiogenesis in DFU are inhibited by IL-1β, TNF-α, and monocyte chemoattractant protein 1, which ultimately interfere with wound healing (117). This study showed that the dosing regimen of BSP affects the local production of TNF-α and IL-1β (especially in skin tissue), excluding serum levels.

BSP reduced macrophage infiltration and increased angiogenesis in cutaneous wound tissue. These results are in line with increased TNF-α and IL-1β levels in skin wound tissue. The potential of BSP to improve diabetic wound healing may be due to its inhibition of NLRP3 inflammasome activation in macrophages. Although the study did not explicitly mention that BSP inhibits the pyroptotic pathway, it is closely related to NLRP3. In addition to promoting the maturation and release of IL-1β, the over-activation of the NLRP3 inflammasome also leads to the over-activation of pyroptosis. In future research, we can consider detecting the expression of ASC-pyroptosome and GSDMD and explore the molecular mechanism of the pyroptosis and inflammasome pathway.

Paeoniflorin is one of the main active components in Paeonia alba Radix, with antioxidant and anti-inflammatory effects (118). The use of paeoniflorin can alleviate diabetic nephropathy by inhibiting the release of inflammatory cytokines and chemokines (TNF-α, IL-1β, and MCP-1) through toll-like receptor 2 (TLR2) inactivation (119). In recent years, traditional Chinese medicine has been recommended as adjuvant therapy for DFU patients (120). Herbal products containing phenolic compounds, terpenoids, or glycosides have positively affected managing diabetic complications (121). Activation of NLRP3 inflammasome in diabetic wounds can maintain inflammation and delay the wound healing process, so inhibiting the activation of NLRP3 inflammasome and the production of IL-1β can effectively promote the healing of diabetic wounds. Paeoniflorin treatment reduced inflammatory cells and decreased the expression levels of NLRP3 and cleaved-caspase-1. In addition, paeoniflorin significantly down-regulated IL-1β, IL-18 and TNF-α levels in DFU.

Paeoniflorin efficiently suppressed NLRP3 and NF-B-mediated DFU inflammation by inhibiting CXCR2, according to *in vitro* findings (122). CXCR2 is a neutrophil receptor that is activated by chemokines like CXCL1 and CXCL2. The activation of the NF-κB pathway in response to IL-1β drives the production of these chemokines. Additionally, studies have shown that TNF-α can regulate the chemokine network in inflammation-related diseases through the NF-κB signaling pathway (123, 124). CXCR2 has critical functions in neutrophil activation and recruitment at inflammatory sites, which provides a reference for positioning CXCR2 as a drug target for many inflammatory diseases. In HG-treated HaCaT cells, blockade of CXCR2 blunts NLPR3/ASC inflammasome activation. In conclusion, paeoniflorin inhibits the formation of NLRP3/ASC/caspase-1 inflammasome and the NF-κB transcription by blocking CXCR2, inhibiting the release of pro-inflammatory cytokines, and promoting the healing of diabetic wounds (96). Although the current evidence is insufficient and further exploration is needed, studies have shown that paeoniflorin is a potential drug for the treatment of DFU.

**Figure 3:**
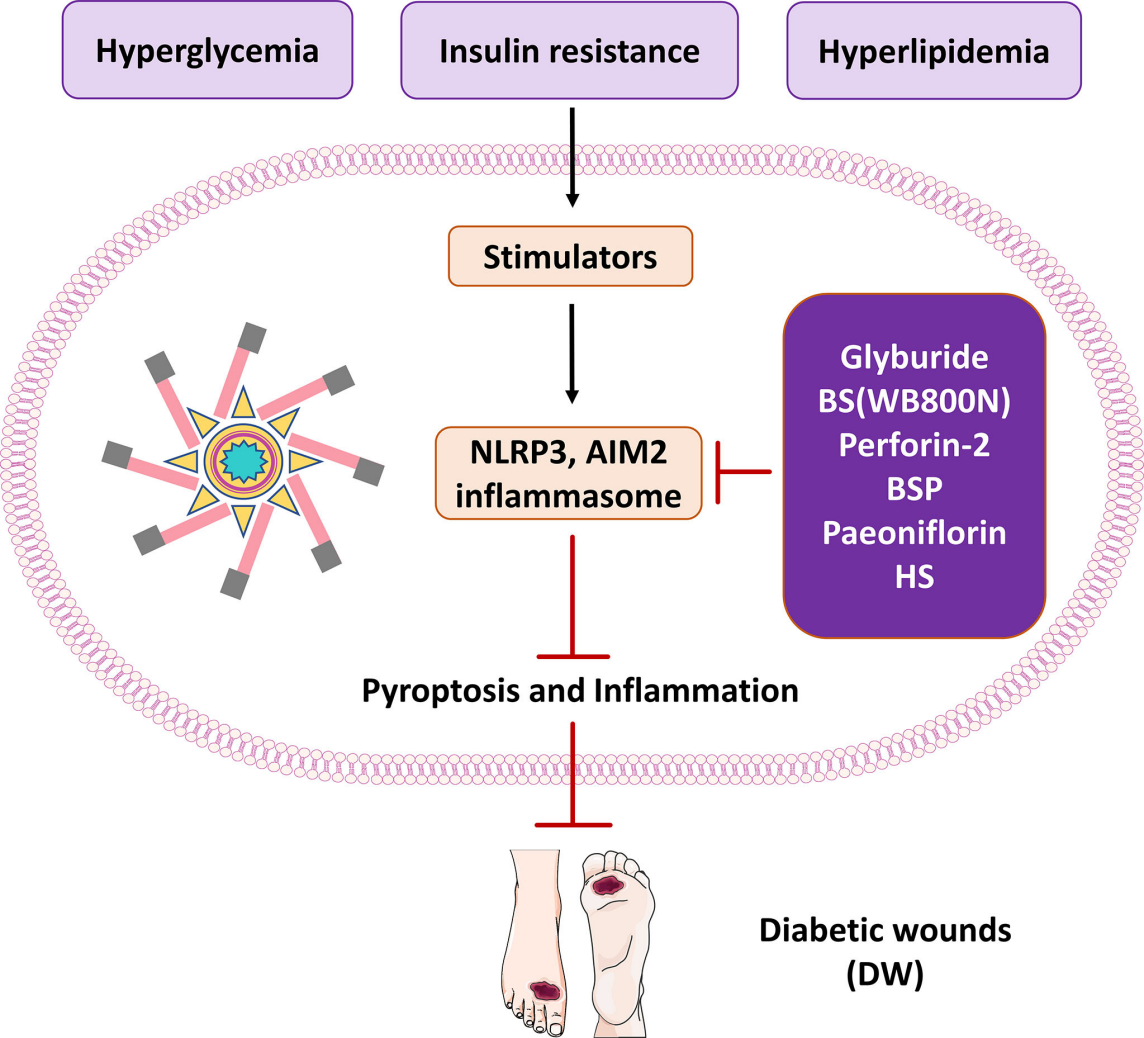
The therapeutic significance of potential molecules or materials for diabetic wound by inhibiting pyroptosis and inflammasome NLRP3 and AIM2.

Heparan sulfate (HS) is a structural element of tissue scaffold and regulates activities of locally synthesized proteolytic enzymes, morphogens, chemokines and growth factors (125, 126). Morphogens are proteins that encode transcription factors, receptors and regulate translation. HS was shown to improve the healing of diabetic wounds in rats by reducing the inflammatory response (127). DAMPs are increased during diabetes to act as activators of the NLRP3 inflammasome and these activators promote inflammasome assembly leading to insulin resistance and organ dysfunction (128). Based on the existing results, it is speculated that HS may promote wound healing by reducing neutrophil infiltration and accumulation of macrophages (129). Moreover, diabetic wounds are more prone to inflammation than different types of wounds. Cleaved-IL-1β and IL-18 play key roles in wound inflammation through interactions with pro-inflammatory cytokines. Research evidence indicates that antagonists of cleaved-IL-1β or IL-18 may be used to treat inflammatory diseases such as gout, arthritis, or arthritic pain. Among them, TNF-α is a representative pro-inflammatory factor, and high levels of TNF-α can amplify and prolong the inflammatory response. The study found that HS significantly reduced Cleaved-IL-1β, IL-18 and TNF-α levels in diabetic rats (130). In addition, it may be possible for HS to work as an anti-inflammatory mechanism by inhibiting the NLRP3 inflammasome activity, so that controlling the level of NLRP3 inflammasome activity can be a method for treating diabetic wounds (97). In the process of wound healing, HS can promote wound healing in diabetic rats. The activation of Cleaved-IL-1β, IL-18 and TNF-α was decreased, and the expressions of NLRP3 and ASC were decreased in the HS group. HS inhibits the inflammatory response and promotes wound healing during diabetic wound healing by down-regulating NLRP3 inflammasome and Cleaved-IL-1β. Therefore, reducing the production of inflammatory factors and the reduction of neutrophil infiltration in the diabetic wound will improve the diabetic wound environment and ultimately shorten the wound healing time (**Figure 3**).

The NLRP3 inflammasome plays an essential physiological role in skin wound healing. Therefore, targeting NLRP3 inflammasome activity and its effectors may be an effective therapeutic strategy to reduce chronic inflammation and promote healing in diabetic wounds. During wound healing, the transient activity of the inflammasome promotes the propagation of wound inflammation and is critical for both epidermis and dermis healing (131). Among the strategies for inhibiting pyroptosis and the NLRP3 inflammasome signaling pathway, drugs such as glyburide and metformin are the closest to clinical translation. In published studies, a variety of compounds can be used to directly or indirectly inhibit NLRP3 inflammasome activity in diabetic wounds. Improves diabetic wound healing by affecting pyroptosis and NLRP3 inflammasome or upstream and downstream signaling. Most researchers are now keen to inhibit the inflammasome pathway to improve diabetic wounds. However, it is often overlooked that inflammation and pyroptosis are closely related. The research horizon should be broadened, and more attention should be paid to pyroptosis while detecting the inflammasome pathway, which may lead to surprising results.

## Conclusion and future prospectives

In recent years, numerous studies have demonstrated that pyroptosis plays a vital role in developing diabetes and its complications. This article has reviewed the impact and role of pyroptosis in diabetic wound healing and the inflammasome is a crucial player in pyroptosis. Danger signals or stimuli cause the activation of caspase-1/4/5/11/3/8 and release IL-1β, IL-18 and other inflammatory factors, resulting in cell pyroptosis. Pyroptosis inhibits wound healing and prolongs the inflammatory response in diabetic wounds, and multiple studies have demonstrated that inhibition of pyroptosis improves wound healing. This review has described some potential drugs and molecules that may be helpful to targets for managing and treating diabetic wounds in the future.

At present, there are few studies on pyroptosis in diabetic wounds, and extensive research is needed to deeply analyze and elucidate the mechanisms and pathophysiological roles of pyroptosis and inflammasome in diabetic wounds. Furthermore, unlike common cell death mechanisms, ferroptosis and cuproptosis have become research hotspots in recent years. Among the forms of cell death in wound healing or diabetic wound healing, the possibility of other forms of cell death (such as necroptosis, ferroptosis, and cuproptosis) remains to be confirmed and is an area worthy of future attention and research. A more in-depth examination of the various modes of cell death could provide new insights into the pathogenesis and development of diabetic wounds.

## Author Contributions

XM was responsible for the literature review and writing. XW, WH and YL were responsible for proofreading. XN and FW were responsible for correction. All authors contributed to the article and approved the submitted version.

## Funding

This work was supported by the National Natural Science Foundation of China (82160770, 81960741, 82060687), the Guizhou Provincial Natural Science Foundation (QKH-J-2020-1Z070), Outstanding Young Scientific and Technological Talents Project of Guizhou Province (2021-5639), *Dendrobium* Specialized Class Project of Guizhou Province (QSKH-2019003).

## Conflict of interest

The authors declare that the research was conducted in the absence of any commercial or financial relationships that could be construed as a potential conflict of interest.

## Publisher’s note

All claims expressed in this article are solely those of the authors and do not necessarily represent those of their affiliated organizations, or those of the publisher, the editors and the reviewers. Any product that may be evaluated in this article, or claim that may be made by its manufacturer, is not guaranteed or endorsed by the publisher.
